# Vascular Proteome Responses Precede Organ Dysfunction in a Murine Model of Staphylococcus aureus Bacteremia

**DOI:** 10.1128/msystems.00395-22

**Published:** 2022-08-01

**Authors:** James T. Sorrentino, Gregory J. Golden, Claire Morris, Chelsea D. Painter, Victor Nizet, Alexandre Rosa Campos, Jeffrey W. Smith, Christofer Karlsson, Johan Malmström, Nathan E. Lewis, Jeffrey D. Esko, Alejandro Gómez Toledo

**Affiliations:** a Bioinformatics and Systems Biology Graduate Program, University of California, San Diego, La Jolla, California, USA; b Department of Bioengineering, University of California, San Diego, La Jolla, California, USA; c Department of Cellular and Molecular Medicine, University of California, San Diego, La Jolla, California, USA; d Glycobiology Research and Training Center, University of California, San Diego, La Jolla, California, USA; e Department of Pediatrics, University of California, San Diego, La Jolla, California, USA; f Skaggs School of Pharmacy and Pharmaceutical Sciences, University of California, San Diego, La Jolla, California, USA; g The Cancer Center and The Inflammatory and Infectious Disease Center, Sanford-Burnham-Prebys Medical Discovery Institute, La Jolla, California, USA; h Department of Clinical Sciences, Division of Infection Medicine, Lund University, BMC, Lund, Sweden; i National Biologics Facility, Technical University of Denmark, Krogens-Lyngby, Denmark; Weill Cornell Medicine-Qatar

**Keywords:** vascular glycocalyx, proteome, sepsis, *Staphylococcus aureus*, DIA mass spectrometry, glycocalyx, vascular

## Abstract

Vascular dysfunction and organ failure are two distinct, albeit highly interconnected, clinical outcomes linked to morbidity and mortality in human sepsis. The mechanisms driving vascular and parenchymal damage are dynamic and display significant molecular cross talk between organs and tissues. Therefore, assessing their individual contribution to disease progression is technically challenging. Here, we hypothesize that dysregulated vascular responses predispose the organism to organ failure. To address this hypothesis, we have evaluated four major organs in a murine model of Staphylococcus aureus sepsis by combining *in vivo* labeling of the endothelial cell surface proteome, data-independent acquisition (DIA) mass spectrometry, and an integrative computational pipeline. The data reveal, with unprecedented depth and throughput, that a septic insult evokes organ-specific proteome responses that are highly compartmentalized, synchronously coordinated, and significantly correlated with the progression of the disease. These responses include abundant vascular shedding, dysregulation of the intrinsic pathway of coagulation, compartmentalization of the acute phase response, and abundant upregulation of glycocalyx components. Vascular cell surface proteome changes were also found to precede bacterial invasion and leukocyte infiltration into the organs, as well as to precede changes in various well-established cellular and biochemical correlates of systemic coagulopathy and tissue dysfunction. Importantly, our data suggest a potential role for the vascular proteome as a determinant of the susceptibility of the organs to undergo failure during sepsis.

**IMPORTANCE** Sepsis is a life-threatening response to infection that results in immune dysregulation, vascular dysfunction, and organ failure. New methods are needed for the identification of diagnostic and therapeutic targets. Here, we took a systems-wide approach using data-independent acquisition (DIA) mass spectrometry to track the progression of bacterial sepsis in the vasculature leading to organ failure. Using a murine model of S. aureus sepsis, we were able to quantify thousands of proteins across the plasma and parenchymal and vascular compartments of multiple organs in a time-resolved fashion. We showcase the profound proteome remodeling triggered by sepsis over time and across these compartments. Importantly, many vascular proteome alterations precede changes in traditional correlates of organ dysfunction, opening a molecular window for the discovery of early markers of sepsis progression.

## INTRODUCTION

Sepsis is a dysregulated host response to infections that can rapidly evolve into a life-threatening pattern of multiple organ dysfunction and is often linked to the presence of bacteria in the bloodstream or bacteremia (1). The disease burden of sepsis has been estimated at ~50 million incident cases and ~11 million fatalities each year, which accounts for almost 25% of all annually reported global deaths (2). Despite these shockingly high levels of morbidity and mortality, no specific sepsis biomarkers are currently available. Treatments remain generic and limited to broad-spectrum antibiotic therapy, intravenous fluid resuscitation, and supportive care (3, 4). The molecular basis of disease progression is also poorly understood, hindering the early recognition and management of critically ill patients in the intensive care units (ICUs).

Vascular dysfunction and organ failure are two highly intertwined clinical outcomes that determine most of the morbidity and mortality of human sepsis (5). Being a systemic syndrome, sepsis is characterized by extensive molecular cross talk between dysregulated inflammatory, coagulopathic and metabolic processes, operating both at the local and systems levels, and compartmentalized across the blood-tissue interfaces of the organs. Therefore, dissecting the individual contribution of vascular dysfunction to the development of organ damage is a difficult task. Nevertheless, understanding the spatiotemporal relationships between vascular and parenchymal tissues at the molecular level might unveil novel ways to impact sepsis outcomes. For example, pharmacological stabilization of the angiopoietin (Ang)/Tie2 axis reduces vasculopathy and inflammation in preclinical models of sepsis, directly implicating endothelial dysfunction in the etiology of organ failure and emphasizing the vasculature as a legitimate therapeutic target (6–8). However, the temporal and causal links between vascular dysfunction and organ damage are still poorly understood, and new methodological frameworks are needed to capture disease progression in a compartmentalized and time-resolved fashion.

Sepsis induces dramatic perturbations in the plasma and organ proteomes of both humans and mice, highlighting the potential of proteomics as a sensitive molecular readout of disease progression (9–14). However, most of the proteomics studies to date are limited to the analysis of single time points and/or single tissue compartments. In fact, despite the recurrent observation that vascular failure lies at the core of sepsis pathology, methodological limitations still preclude the detailed study of vascular proteome changes in sepsis and of their contribution to organ damage. Vascular proteome profiling *in vivo* is particularly challenging, and the suitability of *in vitro* cellular systems has been questioned in multiple studies (15, 16). We have previously developed chemical-biology methods to tag vascular cell surface proteins *in vivo* through the delivery of biotin tags via systemic perfusion. This method uses short perfusion times with ice-cold buffers to preserve tissue integrity, which results in the specific labeling of vascular and perivascular proteins normally accessible to the blood flow, as assessed both by histology and mass spectrometry in uninfected mice as well as in murine models of bacteremia (17, 18). Using this approach, we have observed previously that Staphylococcus aureus sepsis triggers substantial changes in the murine vascular proteome. However, the temporal dynamics of the vascular proteome during infection and its molecular relationships with plasma and extravascular compartments, as well as its contribution to overall sepsis progression, remain unaddressed.

**FIG 1:**
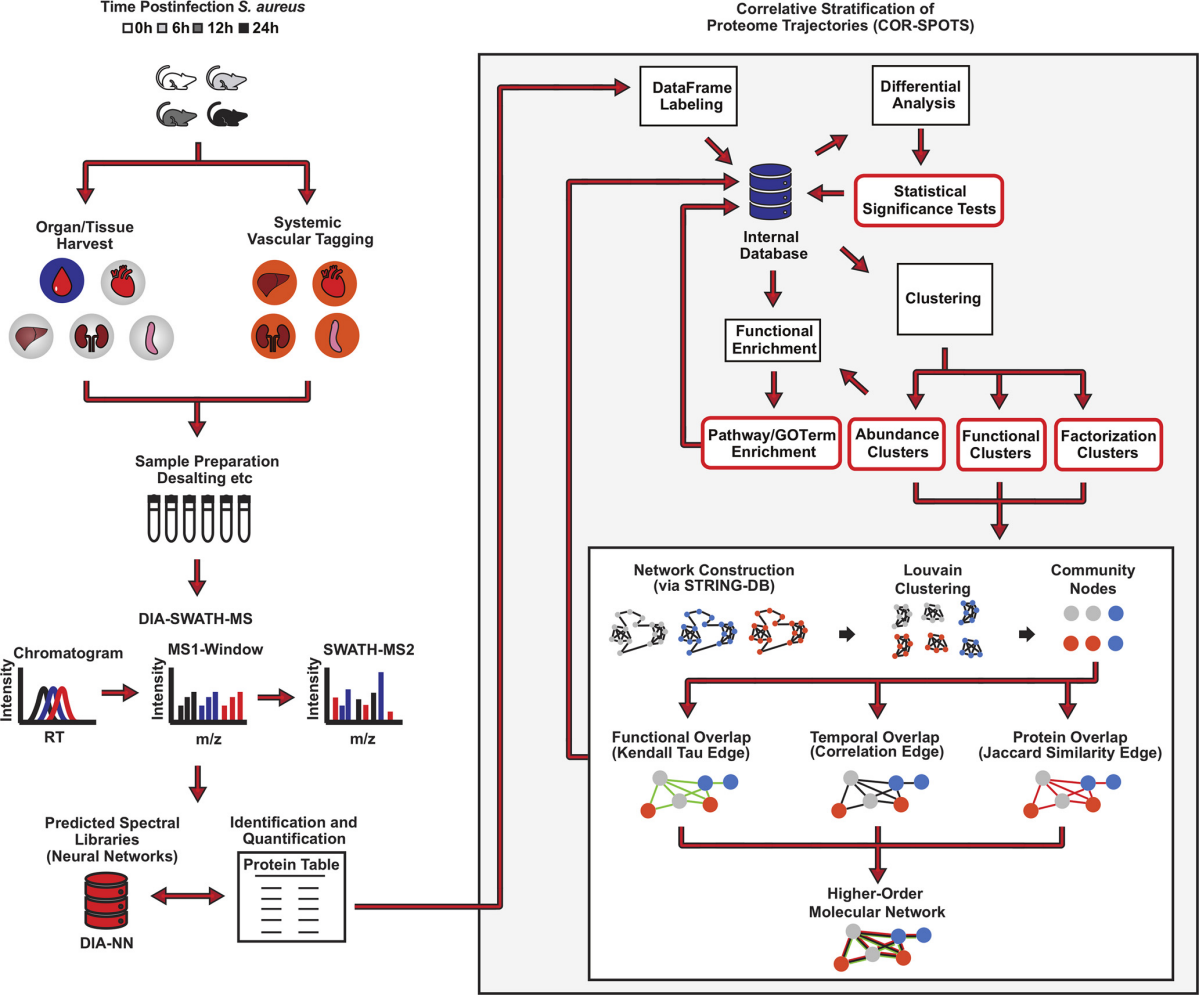
Experimental design and analytical workflow employed in the current study.

In this study, we hypothesize that dysregulated vascular proteome responses predispose the organism to organ failure. To address this hypothesis, we have now experimentally reinterrogated our model of S. aureus sepsis and (i) deployed robust data-independent acquisition (DIA) mass spectrometry workflows to conduct a longitudinal and comprehensive proteomics analysis of the changes triggered by systemic infection across the vascular cell surface proteome of four major organs, (ii) analyzed proteome changes taking place in extravascular tissue compartments and plasma, and (iii) developed a completely novel computational framework to integrate the data across organs, compartments, and time points. Using chemical-proteomics and an integrative computational pipeline, we generated the largest molecular description of the proteome trajectory of sepsis so far, in a compartmentalized and time-resolved fashion. We provide new evidence for the presence of organotypic proteome changes triggered by S. aureus sepsis across the blood-tissue interfaces of four major murine organs (Fig. 1). Novel information generated by these approaches reveals that these proteome responses are largely compartmentalized, temporally coordinated, and significantly correlated with the progression of the disease. Surprisingly, vascular cell surface proteome alterations were found to precede changes in well-established cellular and biochemical correlates of sepsis, opening a completely new window for the discovery of early diagnostic markers of sepsis, as well as novel therapeutic targets. More importantly, our data suggest a potential role for the vascular cell surface proteome as a determinant of the susceptibility of the organs to develop coagulopathy and tissue dysfunction.

## RESULTS

### A two-staged model of the plasma proteome response to sepsis.

**FIG 2:**
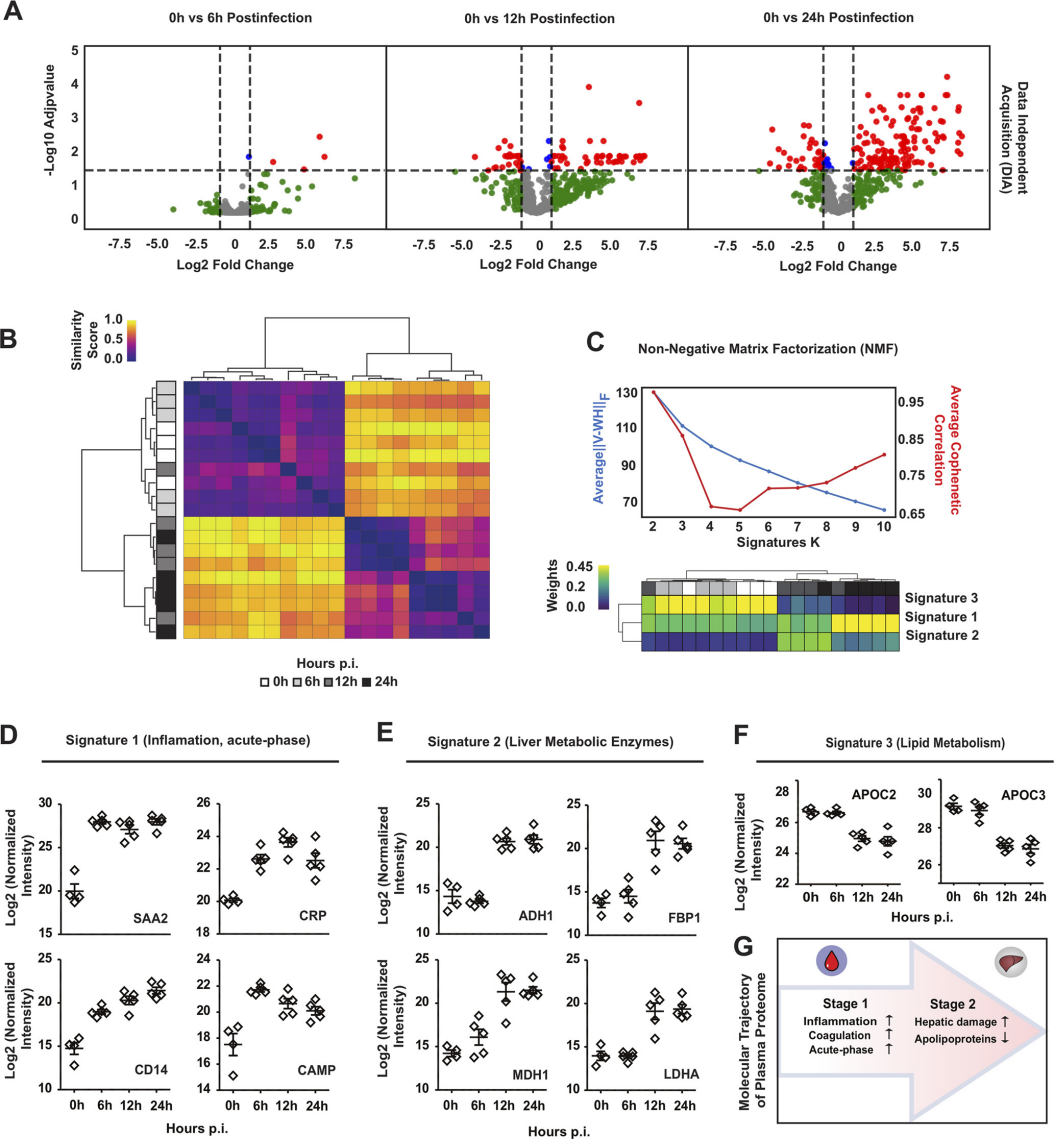
Time-resolved analysis of plasma proteome trajectories in a murine model of Staphylococcus aureus sepsis. (A) Volcano plots showing time-dependent changes in protein abundances in murine plasma at 0 h (*n* = 5), 6 h (*n* = 5), 12 h (*n* = 5), and 24 h (*n* = 5) postinfection and captured by proteomics analysis using data-independent acquisition (DIA) mass spectrometry. Coloring thresholds were set at a fold change of >2 and *P* value of <0.05. (B) Hierarchical clustering of the time-course plasma samples resolved by the DIA-SWATH-MS proteomic analysis. (C) Nonnegative matrix factorization (NMF) analysis of the plasma proteome changes converges into three major protein signatures with distinct temporal behavior. The top graph depicts the model fit for the NMF computed at k = 2 to 10 across 200 random initializations. The bottom heatmap represents the distribution of the three major protein signatures selected from the top scoring model (k = 3). (D to F) Representative normalized intensity values for the top proteins in the NMF signature 1 (D), signature 2 (E), and signature 3 (F). (G) Schematic depiction of the two-staged model of the plasma proteome trajectory over the course of infection.

To start tracking the progression of the host response to S. aureus bacteremia, mice were challenged intravenously with a 100% lethal dose (LD_100_) of bacteria, and their plasma proteomes were analyzed at 0 h, 6 h, 12 h, and 24 h postinfection. To facilitate accurate time-resolved quantitative proteomics, a DIA workflow based on sequential window acquisition of all theoretical mass spectra (SWATH-MS) was applied (19). DIA-SWATH-MS uses broad isolation windows to measure almost all MS-detectable peptides in a biological sample. This workflow results in an increased number of protein identifications and more robust quantification than approaches based on data-dependent acquisition (DDA). Initially, 1 μL of nondepleted unfractionated plasma from S. aureus-infected or healthy control mice was subjected to proteomics analysis. Protein intensities were extracted from the DIA data using predicted spectral libraries generated through deep neural networks in the software suite DIA-NN (20) and further constrained by experimental information from public databases (Materials and Methods). As shown in Fig. 2A, the plasma proteome is significantly altered during infection, and clustering analysis segregates the samples into early and late responses (Fig. 2B; see Data Set S2 in the supplemental material). To uncover the main protein changes driving the partitioning of the samples, the data were deconvolved through nonnegative matrix factorization (NMF) (21). Three NMF clusters or “protein signatures” were successfully identified and condensed into discrete proteome trajectories (i.e., temporal intensity patterns) (Fig. 2C). The NMF signature 1 identified the early (6 h) accumulation of inflammatory proteins and acute-phase reactants (APRs), including the C-reactive protein (CRP), the serum amyloid protein 2 (SAA2), and soluble CD14, as well as antimicrobial factors from the cathelicidin family (Fig. 2D). The NMF signature 2 was characterized by the plasma accumulation of liver metabolic enzymes (Fig. 2E), whereas the NMF signature 3 identified the depletion of hepatic factors involved in lipid metabolism (Fig. 2F). These two last signatures were linked to the late time points (12 h and 24 h) and were indicative of the occurrence of acute liver failure. Based on these data-driven patterns, the plasma proteome analysis reflected a two-staged model of the progression of sepsis, with each stage associated with specific inflammatory and metabolic alterations. (Fig. 2G).

10.1128/msystems.00395-22.5 DATA SET S2 Normalized protein intensities for plasma and parenchymal proteomes. The whole quantified proteome for plasma, liver, kidney, heart, and spleen. Both intensities harvested from the DDA and DIA approaches are listed for comparison. Download Data Set S2, XLSX file, 2.1 MB. Copyright © 2022 Sorrentino et al. 2022 Sorrentino et al. https://creativecommons.org/licenses/by/4.0/ This content is distributed under the terms of the Creative Commons Attribution 4.0 International license.

### Vascular cell surface proteome responses are temporally coordinated in an organ-specific fashion.

**FIG 3:**
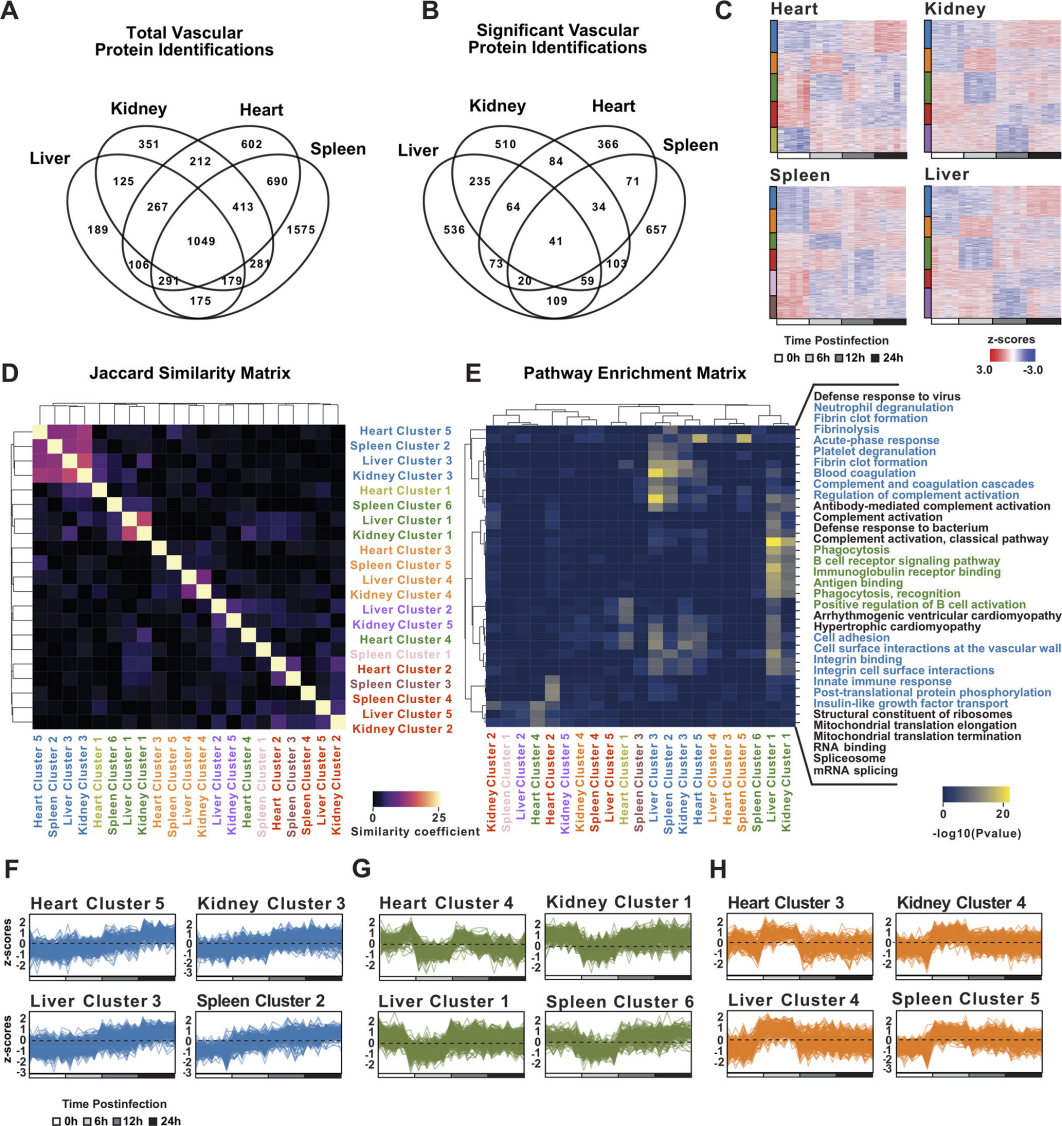
Coordinated vascular proteome responses triggered by S. aureus sepsis. (A) Total number of proteins identified through systemic vascular tagging across liver, kidney, heart, and spleen at 0 h, 6 h, 12 h, and 24 h postinfection (*n* = 4 to 5 per group). (B) Significantly altered proteins using a one-way ANOVA (FDR, <0.10) across the four organs and the intersections between them. (C) Heatmaps showing the k-means clustering of significant protein identifications in the heart, kidney, spleen, and liver. Clusters with the same proteome trajectory (i.e., temporal intensity patterns) are coded with the same color as represented in the bars to the left of the heatmaps. (D) Jaccard similarity matrix (intersection over the union) representing the percent overlap of the protein identifications across vascular clusters. Blue and green squares refer to subsets of vascular clusters displaying a high overlap of proteins, and the orange squares represent a subset of vascular clusters displaying low overlap. All cluster colors are according to the proteome trajectories. (E) Hierarchical clustering of the main biological pathways obtained from functional enrichment analysis of the vascular clusters colored according to *P* value. (F to H) Trace representations of the normalized protein abundances (Z-scores) for the vascular clusters that increase over time (F), decrease at 6 h (G), or increase at 6 h postinfection (H).

Vascular surface proteins are located at the blood-tissue interface of the organs, which makes them potential targets of early molecular perturbations induced by blood infections. To examine whether sepsis triggers a similar staging pattern in the vascular proteome as observed in the plasma proteome, infected mice underwent systemic perfusion using sulfo-*N*-hydroxysuccinimide (NHS)-biotin to tag proteins located on the vascular surfaces (17). Four major organs were selected for the analysis (liver, kidney, heart, and spleen), based on their clinical significance, their high level of vascularization, and the observation of specific pathological changes taking place across these tissues in the model of S. aureus sepsis, as reported previously (17). This technique greatly enriches for proteins accessible to the blood, with very little penetration into the parenchyma based on streptavidin staining of tissues (17). Biotinylated proteins were enriched on streptavidin columns and analyzed by DIA-SWATH-MS. Thousands of proteins were quantified accurately across the vasculature of the organs (Fig. 3A), resulting in a substantial increase in the number of identifications compared with our previous studies using regular shotgun proteomics (see Fig. S1A to C and Data Set S1 in the supplemental material). Additionally, a statistical analysis indicated that a large fraction of the vascular cell surface proteome was significantly perturbed during infection (Fig. 3B). Clustering of the data shows that the septic vascular cell surface proteome has a more complex kinetics than the plasma proteome, displaying clusters with similar temporal behaviors across the organs, and protein clusters exhibiting organ-specific changes (Fig. 3C). Cluster clarity was further assessed using a silhouette analysis (Fig. S1D).

10.1128/msystems.00395-22.1 FIG S1 Comparison of the vascular cell surface proteins identified previously using a standard shotgun DDA-proteomics workflow (17) and in this study using a DIA-SWATH-MS pipeline. Additional cluster clarity assessment of the vascular cell surface proteins. (A to C) Venn diagrams showing unique and shared protein identifications in liver, kidney, and heart, respectively. (D) Silhouette analysis providing graphical representation of how well each cell surface protein has been classified into k clusters. The silhouette value is a measure of how similar an object is to its own cluster compared with other clusters. Download FIG S1, TIF file, 1.5 MB. Copyright © 2022 Sorrentino et al. 2022 Sorrentino et al. https://creativecommons.org/licenses/by/4.0/ This content is distributed under the terms of the Creative Commons Attribution 4.0 International license.

10.1128/msystems.00395-22.4 DATA SET S1 Normalized protein intensities for enriched vascular proteomes. The whole quantified proteome for the enriched liver, kidney, heart, and spleen vasculature. Download Data Set S1, XLSX file, 1.6 MB. Copyright © 2022 Sorrentino et al. 2022 Sorrentino et al. https://creativecommons.org/licenses/by/4.0/ This content is distributed under the terms of the Creative Commons Attribution 4.0 International license.

To determine if vascular cell surface proteome trajectories shared across the organs reflect the regulation of similar proteins, all identified proteins were clustered and all vascular clusters were extracted, and both their compositions, similarity coefficient, and functional enrichment were evaluated. The objective was to identify groups of proteins with distinct kinetic behaviors over the time of infection and link them to specific biological processes. Four clusters (heart cluster-5, spleen cluster-2, liver cluster-3, and kidney cluster-3) (Fig. 3D, blue) shared a significant number of proteins (~15%) and common biological pathways, including neutrophil and platelet degranulation, activation of coagulation and complement, and regulation of leukocyte-endothelial interactions (Fig. 3D and E). The abundance of all proteins in these clusters was linearly increased over the time of infection (Fig. 3F). Additionally, three other clusters (spleen cluster-6, liver cluster-1, and kidney cluster-1) (Fig. 3D, green) also shared many proteins (~14%), as well as pathways related to phagocytosis and the adaptive immune response, particularly receptor-mediated interactions (Fig. 3E and G). Interestingly, the abundance of all proteins in these clusters dropped dramatically at 6 h postinfection, suggesting the rapid downregulation and/or shedding of a large fraction of the vascular proteome. We mapped these downregulated proteins to the plasma compartment and found that the levels of some of them but not all were increased simultaneously in plasma, suggesting a complex interaction between tissue source, shedding/downregulation, and systemic clearance (see Fig. S2 in the supplemental material). In sharp contrast to these general responses across the organs, evidence of tissue-specific changes was also detected. For example, some specific clusters (heart cluster-3, spleen cluster-5, liver cluster-4, and kidney cluster-4) (Fig. 3D, orange) shared only a few proteins (~2%), suggesting the induction of organotypic responses. Functional enrichment linked these clusters to ion transport and homeostasis (heart cluster-3), metal sequestration by antimicrobial proteins (spleen cluster-5), cellular responses to labile heme (liver cluster-4), and mitochondrial dysfunction (kidney cluster-4). Notably, despite the marked differences in their protein contents, these clusters exhibited synchronous proteome trajectories (Fig. 3H). Taken together, vascular responses to sepsis entail both general and organotypic alterations of the vascular proteome very early during disease progression.

10.1128/msystems.00395-22.2 FIG S2 Plasma levels of proteins downregulated at 6 h postinfection in the vascular surfaces. The proteins that were significantly downregulated at 6 h postinfection in the vascular surface proteomes of the 4 organs (Fig. 3G, green traces, total of 647 proteins) were mapped to the data set containing the proteins significantly dysregulated in plasma. This map resulted in 88 proteins displaying synchronously changes in systemic circulation. Download FIG S2, TIF file, 1.8 MB. Copyright © 2022 Sorrentino et al. 2022 Sorrentino et al. https://creativecommons.org/licenses/by/4.0/ This content is distributed under the terms of the Creative Commons Attribution 4.0 International license.

### Comparison between parenchymal and vascular cell surface proteome alterations during sepsis.

**FIG 4:**
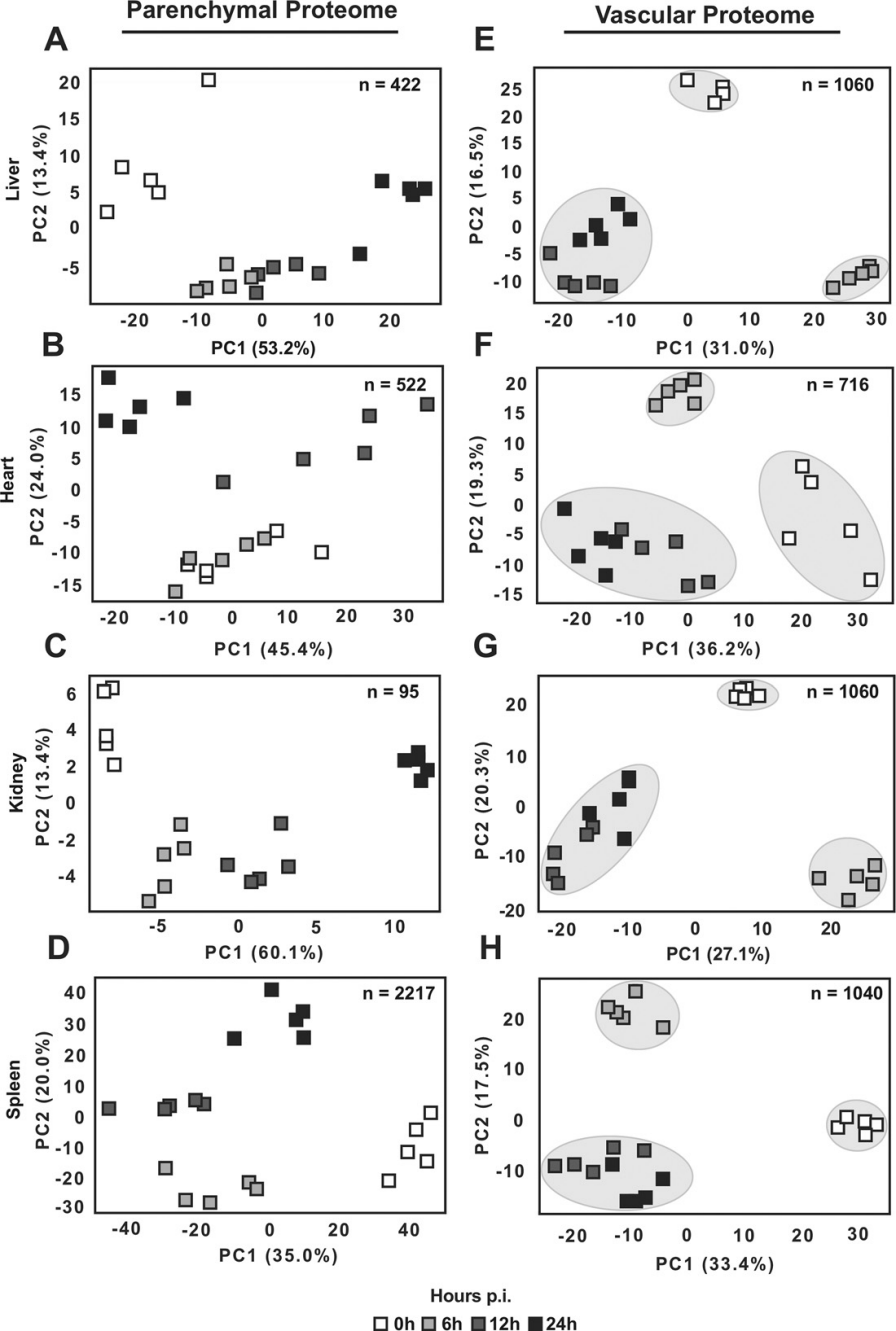
Principal-component analysis (PCA) of significantly altered parenchymal and vascular proteins during infection. (A to H) Proteins identified displaying statistically significant temporal patterns using a one-way ANOVA (FDR, <0.10) were subject to PCA analysis to identify time-dependent variability for liver parenchymal proteins (*n* = 422) (A), heart parenchymal proteins (*n* = 522) (B), kidney parenchymal proteins (*n* = 95) (C), spleen parenchymal proteins (*n* = 2,217) (D), liver vascular proteins (*n* = 1,060) (E), heart vascular proteins (*n* = 716) (F), kidney vascular proteins (*n* = 1,060) (G), and spleen vascular proteins (*n* = 1,040) (H).

To determine the progression of parenchymal proteome responses, infected organs were subjected to whole-tissue proteomics analysis, which resulted in the accurate quantification of thousands of proteins (Data Set S2). Like the vascular proteome, parenchymal responses displayed both shared and organ-specific proteome trajectories (see Fig. S3 in the supplemental material). The principal-component analysis (PCA) shows that parenchymal proteome responses were highly heterogeneous across the organs, displaying different temporal patterns (Fig. 4A to D; see Data Set S3 in the supplemental material). For example, the liver tissue was governed by early changes and a significant separation of the samples at 0 h and 6 h (Fig. 4A), whereas the heart exhibited a more delayed response (no notable difference between 0 h and 6 h, but clear differences between samples at 6 h and 12 h) (Fig. 4B). The kidney and the spleen were also quick responders, although the spleen showed the largest parenchymal proteome changes of all organs, with >2,000 proteins significantly altered during sepsis (Fig. 4C and D). Interestingly, the analysis of the vascular proteomes shows a rather uniform pattern (Fig. 4E to H), in contrast to the heterogeneity of the temporal patterns of the parenchymal responses. All vascular proteome data sets displayed a distinct separation of the 0-h and 6-h time points and clustering of the 12-h and 24-h time points, independently of the organ. Despite this similar behavior, the number of dysregulated proteins that were shared across the organ vascular fractions at 6 h was very low (41, ~1.4%) (Fig. 3B). These findings suggest that sepsis induces early changes in the vascular proteome of all organs, but each proteome response is most likely shaped by organ-specific factors.

10.1128/msystems.00395-22.3 FIG S3 Coordinated parenchymal proteome responses triggered by S. aureus sepsis. (A) Heatmaps showing the k-means clustering of significant (one-way ANOVA, FDR of <0.10) parenchymal protein identifications in the heart (*n* = 5), kidney (*n* = 5), spleen (*n* = 5), and liver (*n* = 5). (B) Jaccard similarity matrix (intersection over the union) representing the overlap of the protein identifications across parenchymal clusters derived from the proteomics analysis. (C) Functional enrichment analysis of the parenchymal proteome clusters. Hypergeometric test for functional enrichment was used and colored according to the *P* value. Hierarchical clustering was utilized to visualize functional similarities between the indicated protein clusters. Clusters with the same proteome trajectory (i.e., temporal intensity patterns) are coded with the same color as represented in the bars to the left of the heatmaps. Download FIG S3, TIF file, 1.9 MB. Copyright © 2022 Sorrentino et al. 2022 Sorrentino et al. https://creativecommons.org/licenses/by/4.0/ This content is distributed under the terms of the Creative Commons Attribution 4.0 International license.

10.1128/msystems.00395-22.6 DATA SET S3 PCA loadings for the proteins contributing to the different components in Fig. 4. Download Data Set S3, XLSX file, 1.0 MB. Copyright © 2022 Sorrentino et al. 2022 Sorrentino et al. https://creativecommons.org/licenses/by/4.0/ This content is distributed under the terms of the Creative Commons Attribution 4.0 International license.

### Building the proteome trajectory of sepsis through a higher-order networking approach.

**FIG 5:**
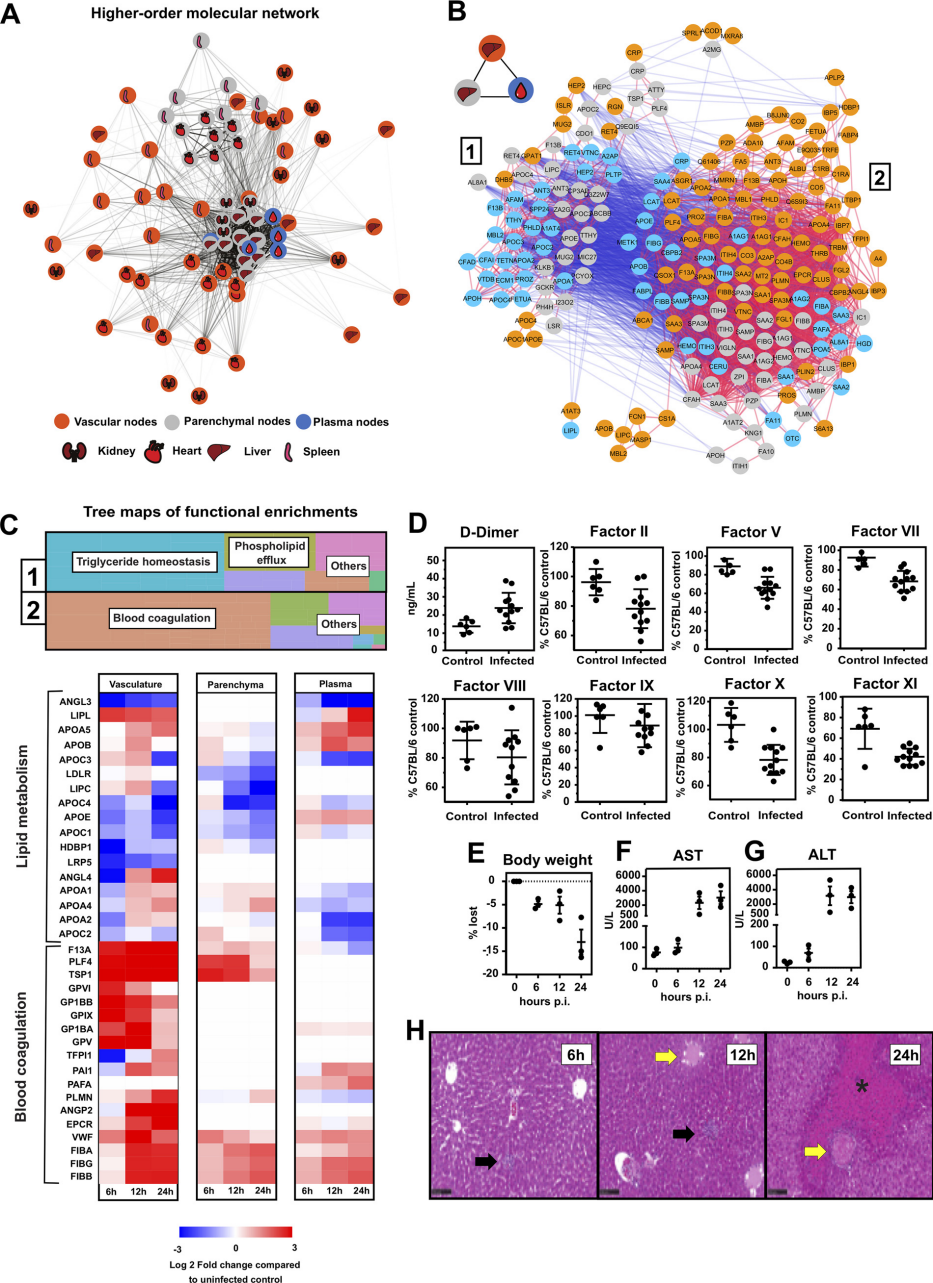
Higher-order molecular networking reveals vascular proteome perturbations preceding coagulopathy and hepatic damage. (A) Complete network analysis of all proteome changes across organs and tissue compartments visualized by the edge-weighted spring embedded layout, where the edge distance and shade represent the proportion of significant pairwise correlations between two nodes. Each node color represents the compartment of origin with tissue represented by gray fill color, vascular represented by orange fill color, and plasma represented by blue fill color. (B) Functional cross talk between liver parenchymal, vascular, and plasma proteins. Three intercorrelated higher-order nodes were selected, and their pairwise protein correlations were extracted. The subnetwork contains 2 major anticorrelated clusters of proteins. Edges are colored using a blue, white, and red continuous scale ranging from −1.0 to 1.0. (C) Functional enrichment analysis of the 2 major protein clusters visualized by treemaps (top). Perturbation of lipid metabolic proteins and upregulation of blood coagulation components are represented by compartmentalized heatmaps of the liver parenchymal tissue, vasculature, and blood plasma. Log_2_-fold change values are compared with uninfected proteome measurements (0 h) for each compartment (bottom). (D) Enzyme activity assays measuring blood coagulation factors present in murine plasma at 24 h postinfection. (E to G) Changes in body weight (E), AST (F), and ALT (G). (H) Representative images of the hematoxylin and eosin stain of liver tissue over the time course. Leukocyte infiltration was noticed as early as 6 h postinfection (black arrows) and vascular occlusion and thrombosis as early as 12 h postinfection (yellow arrows); necrotic areas are marked with an asterisk (*).

To reconstruct the disease trajectory of sepsis using the host proteome response as a readout, we developed a novel computational pipeline to integrate the data across time points, organs, and tissue compartments. Correlative stratification of proteome trajectories (COR-SPOTS) was designed as a higher-order networking approach to store, organize, and integrate multiple data layers based on temporal and functional relationships between the data points (see Materials and Methods). Significantly dysregulated proteins from each organ and compartment (plasma, vascular, and parenchymal) were parsed through STRING-DB to group proteins based on functional associations (22). Highly interconnected clusters of nodes were then identified through the Louvain algorithm for community detection and collapsed into new single nodes. We then computed ~24 million pairwise Spearman correlations over all combinations of detected community nodes, which resulted in ~3.7 million significant correlations (false discovery rate [FDR], <0.10) that were used to define the linking edges of the network. Approximately an equal number of significant positive and negative correlations were observed. Other similarity relationships between the nodes such as the content overlap and the functional enrichment were also stored within the network, resulting in a compiled and fully searchable small database containing all proteomics results and metadata from this study, which can be found on the online repository the Network Data Exchange (NDEx) with universally unique identifier (UUID) 45474980-9d56-11eb-9e72-0ac135e8bacf (Fig. 5A).

### Vascular cell surface proteome responses in the liver precede coagulopathy and hepatic damage.

We have shown previously that the murine model of S. aureus sepsis suffers from a dominant coagulopathic phenotype in the liver (17). To demonstrate the utility of COR-SPOTS to dissect molecular changes across the blood-tissue interfaces, we ranked the nodes and selected the hepatic vascular, parenchymal, and plasma nodes with the highest intercorrelation. Proteins stored within these highly correlated nodes displayed a bimodal network topology characterized by the presence of two distinct anticorrelated clusters of proteins (Fig. 5B). Cluster 1 contained most of the plasma and parenchymal proteins linked to triglyceride and phospholipid metabolism, whereas cluster 2 was enriched in vascular proteins linked to coagulation (Fig. 5C). Many proteins involved in lipid metabolism were diminished rapidly from the hepatic vasculature, including multiple apolipoproteins, angiopoietin-like proteins, and the glycosylphosphatidylinositol-anchored high-density lipoprotein-binding protein 1 (HDBP1). Some of these factors were also reduced in the liver parenchyma and plasma during infection, but these changes were more delayed than the vascular proteome. On the other hand, proteins involved in coagulation were accumulated rapidly in the hepatic vasculature. This finding included the deposition of several platelet glycoproteins (GPIX, GP1BB, GP1BA, and GPV), indicating abundant intravascular platelet deposition, together with an increase in the levels of von Willebrand factor (VWF) and fibrinogen. Most of these changes were specific to the vascular proteome and were captured only weakly in the parenchyma and plasma compartments toward the later time points (12 h and 24 h). Notably, the levels of Tissue Factor Pathway Inhibitor 1 (TFPI1) at 6 h postinfection were depleted rapidly, strongly suggesting a prothrombotic priming of the liver vasculature very early during infection (Fig. 5C). Further confirming that S. aureus bacteremia results in systemic coagulopathy, the levels of d-dimers in plasma were significantly increased at 24 h postinfection, and the widespread consumption of multiple coagulation factors was also confirmed by enzyme activity assays (Fig. 5D). Additionally, most animals lost significant amounts of weight over time due to metabolic alterations and/or changes in body fluid distribution (Fig. 5E). Interestingly, organ failure based on the increased levels of aspartate transaminase (AST) and alanine aminotransferase (ALT) in circulation was not apparent until 12 h postinfection (Fig. 5F and G), which coincided with the occurrence of massive thrombosis and tissue necrosis in the liver toward the late time points (Fig. 5H). Taken together, the data suggest a global reprogramming of the liver by shutting down metabolic functions as the level of intravascular coagulation and vasculopathy increases. More importantly, these global changes were preceded by early alterations of the vascular cell surface proteome, long before signs of organ failure were detected through well-established correlates of organ damage.

### Vascular cell surface proteome responses in the heart precede bacterial invasion and neutrophil infiltration.

**FIG 6:**
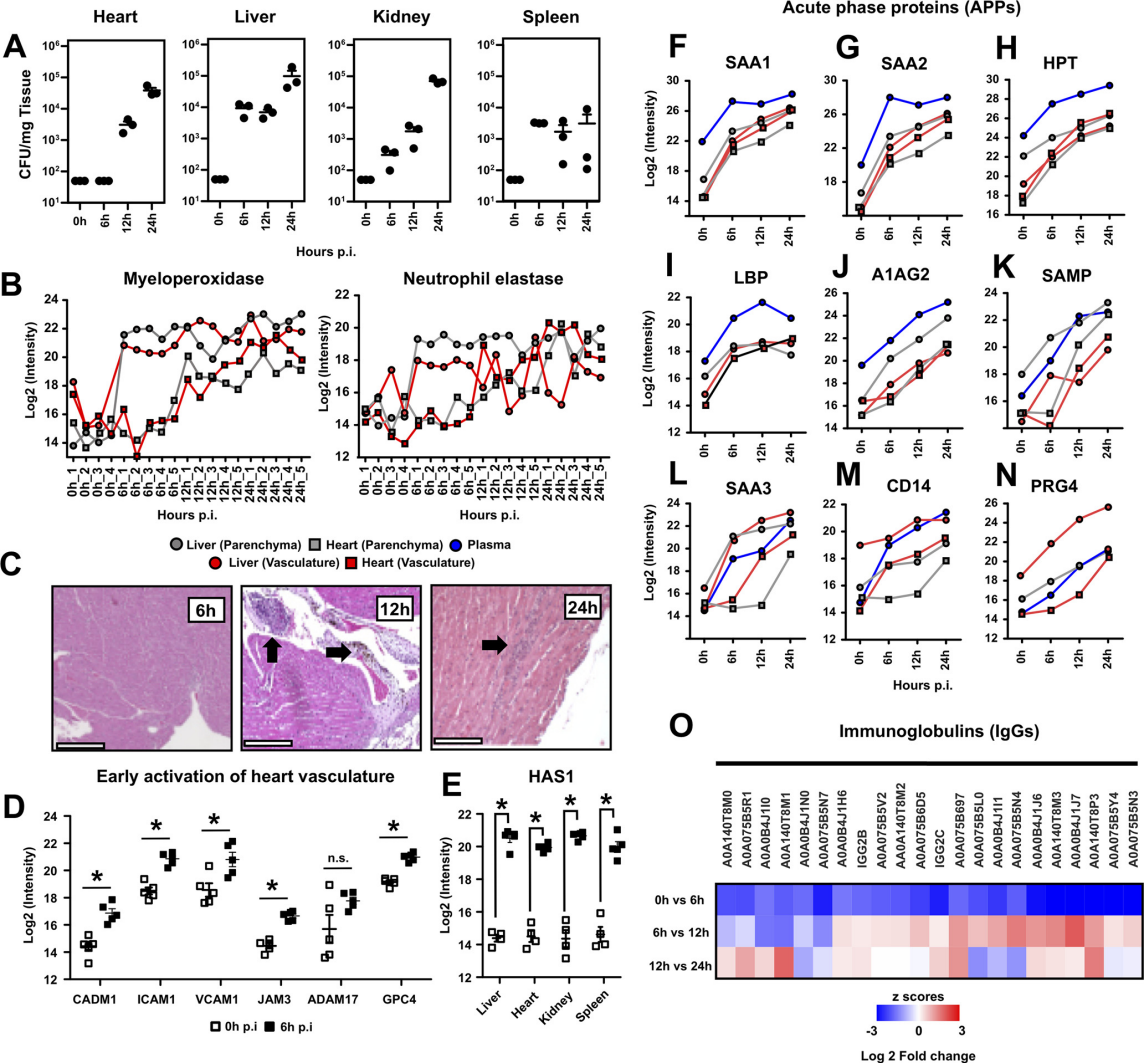
Bacterial invasion and neutrophil infiltration are delayed in the heart compared with other organs. (A) Organs were harvested, homogenized, and plated to quantify the bacterial CFU present over the experiment time course. (B) Proteomic measurements of myeloperoxidase (MPO) and neutrophil elastase (ELANE) for both liver and heart vascular and parenchymal proteomes. Liver measurements are represented by filled circles; heart measurements are represented by filled squares. Tissue was represented by a gray fill color, and vascular measurements by orange fill color. (C) Representative images of histological analysis of heart tissue over the experiment time course. Bacterial and leukocyte infiltration was first noticed at 12 h and 24 h postinfection (black arrows). (D) Cardiac vascular proteins change very early during infection. p.i, postinfection; n.s., not significant. (E) Hyaluronan synthase 1 (HAS1) was significantly increased in the vascular surface of all four organs. (F to N) Global versus compartmentalized changes in the levels of acute phase reactants (APRs) in liver, heart, and plasma. Plot labeling is as described in B, with the addition of filled blue circles for plasma measurements. (O) A total of 23 immunoglobulin proteins were found to dramatically decrease in abundance as early as 6 h postinfection. Log_2_-fold change values are compared with the previous time point.

A comparison between the changes induced by sepsis in the vascular and whole-organ proteomes indicated a delayed response of the cardiac parenchyma compared with the rest of the organs (Fig. 4B). Inspection of the cardiac nodes in the network showed a distinct separation of the parenchymal nodes from the vascular nodes, distinguishing the heart from the other organ proteomes (Fig. 5A). We hypothesize that this disparity in the cardiac protein response was linked to a difference in the kinetics of the disease trajectory of sepsis in the heart and perhaps linked to the presence or absence of major cellular events, such as bacterial invasion and/or leukocyte infiltration of the organs. The bacterial burden of the organs was in line with this hypothesis, showing that despite the presence of bacteria in the liver, kidney, and spleen already by 6 h postinfection, no bacteria were found in the heart until 12 h after inoculation (Fig. 6A). Since neutrophils are the primary responders to the presence of bacterial infiltrates in the tissues, we assessed the levels of myeloperoxidase (MPO) and neutrophil elastase (ELANE), which are two granule proteins that are specific to neutrophil degranulation and formation of extracellular traps. The levels of MPO and ELANE in the liver were raised rapidly upon infection both in the parenchyma and vascular proteomes, whereas their deposition in the cardiac parenchyma and vasculature was first observed after 12 h (Fig. 6B). A histological analysis confirmed that neither bacterial microabscesses nor immune infiltrates were observable in heart tissue until 12 h postinfection (Fig. 6C). Taken together, these data suggest that in this model of S. aureus sepsis, bacterial dissemination and leukocyte infiltration are delayed temporally in the heart compared with those in the other organs, opening an interesting window to capture very early molecular changes in the vasculature as a response to systemic inflammation.

Next, we focused on the changes in the heart vasculature at 6 h to identify molecular alterations that precede the bacterial and leukocyte invasion of the tissue (Fig. 6D). Multiple vascular adhesion proteins were induced rapidly during infection, such as CADM1, ICAM1, and VCAM1. Upregulation of junctional endothelial proteins, such as JAM3, was also observed, together with an increase in the levels of ADAM17, a protease that has been linked to most of the early shedding of the vascular surfaces upon inflammation. Changes in the glycocalyx were also noticed, including upregulation of heparan sulfate proteoglycans, such as GPC4, and very robust induction of the hyaluronic acid synthase HAS1. In fact, early upregulation of HAS1 was observed consistently across all vascular surfaces, directly implicating HAS1 in glycocalyx remodeling during sepsis (Fig. 6E).

### Compartmentalization of acute-phase reactants and immunoglobulin shedding are early vascular responses to S. aureus sepsis.

Acute phase reactants (APRs) are inflammation markers that exhibit rapid and substantial changes in serum concentration during inflammation. Most APRs are synthesized in the liver as a response to both local and systemic inflammation, and some of them get associated with the endothelium, where they exert multiple functions. We used COR-SPOTS to map the dynamics of APRs during infection across organs and compartments. Although the dynamics of the APRs in plasma followed a linear increase, as has been described previously, when mapping the levels of APRs associated with parenchymal and vascular compartments, we noticed at least two distinct patterns. Type I APRs experienced rapid induction in all organs and compartments and accumulated steadily over time. This pattern was characteristic of APRs, such as SAA1, SAA2, HPT, and LBP (Fig. 6F to I). However, we also identified a second group or type II APRs, whose levels were compartmentalized and sensitive to the disease status of the organs. For example, A1AG2 and SAMP levels in liver parenchyma and vascular fractions followed the same kinetics as in plasma (Fig. 6J and K). Interestingly, their levels in cardiac parenchyma and vasculature remained unchanged at 6 h, followed by a linear increase at 12 h and 24 h. Other APRs, such as SAA3, followed a similar trend, except for a temporal difference between the cardiac vascular proteome response (up at 12 h) and the cardiac parenchymal response (up at 24 h) (Fig. 6L). CD14 was also increased in the liver and in the heart vasculature by 6 h, but increased cardiac parenchymal levels were detected only by 24 h (Fig. 6M). We have shown previously that PRG4, a protein that behaves as an APR in many inflammatory models, gets deposited in the liver vasculature in this sepsis model of S. aureus bacteremia by 24 h. Looking at the dynamics of PRG4 across organs and compartments, it became apparent that vascular accumulation starts early in the liver and increases linearly, but an association with the heart vasculature could not be detected until the late time points, while PRG4 remained undetected in the heart parenchyma at all investigated time points (Fig. 6N). Finally, we also observed that in contrast to the increasing levels of many vascular adhesion molecules and APRs, the most dramatic downregulation taken place in the cardiac vasculature at 6 h was the shedding of many immunoglobulins (IgGs), probably loosely associated with the vascular wall, without changing IgG total plasma levels (Fig. 6O). All these changes are consistent with a massive remodeling of the vascular proteome during sepsis, even before bacteria or infiltrating immune cells are observed. These changes are expected to significantly alter the physicochemical properties of the vascular surfaces and their organotypic responses to systemic infection.

## DISCUSSION

Understanding the relationships between vascular failure and organ damage has important implications for sepsis therapeutics and diagnostics. Even though many patients respond quickly to standard care, an unacceptably high fraction of cases still progress into intractable shock responses with a high risk for long-term morbidity and deadly outcomes. Vascular stabilization as a therapeutic goal in sepsis has been discussed for years, although few preclinical studies have been conducted to assess the impact of vasculoprotective approaches on relevant sepsis outcomes, and so far, none of them have succeeded in making a translational jump from the bench to the bedside. Part of the problem lies on the enormous heterogeneity of the organ vasculatures and a lack of detailed knowledge on the molecular composition of the vascular surfaces and how they are affected by sepsis.

Here, we demonstrate that sepsis triggers a series of well-coordinated proteome changes across the blood-tissue interfaces of the organs, but the nature of those changes and their specific temporal patterns are largely organotypic. These organ-specific patterns seem to be linked partially to the occurrence of major sepsis “checkpoints,” such as systemic vascular activation or the actual invasion of the organs by pathogens and infiltrating immune cells. Interestingly, the liver undergoes a rapid reprogramming of its vascular proteome by downregulating lipid metabolic functions and becoming primed toward a prothrombotic and antifibrinolytic state, long before signs of organ dysfunction and actual thrombosis are observable. This finding is remarkable, first because it suggests that vascular proteome alterations can have a yet unrealized potential as a source of early diagnostic and prognostic markers of sepsis. Second, it also opens the possibility that timely therapeutic interventions targeting the vasculature might prevent irreparable organ damage. For example, we observed that hepatic thrombosis is preceded by early depletion of vascular TFPI, upregulation of VWF, and deposition of intravascular platelets. Alterations in the extrinsic pathway of coagulation were also reported in baboon models of Gram-negative sepsis, and exogenous TFPI administration rescues the organs from coagulation-induced damage (23, 24). However, there can be difficulties in translating some of the findings from preclinical models to therapy (25, 26). For example, results from clinical trials to evaluate the efficacy and safety of tifacogin (recombinant TFPI) to treat sepsis have so far been disappointing, emphasizing the complexity of the host response to systemic infection.

The host response to sepsis is overwhelmingly complex since it encompasses cross talk between multiple systems and a time-dependent staging, where both pro- and anti-inflammatory components might be operating at different time points. Predicting the disease trajectory of sepsis is therefore a most sought-after clinical goal. Here, we show that the host proteome response to sepsis is a powerful molecular readout of the septic response. Using a novel networking approach, COR-SPOTS, we were able to identify very early vascular changes that even precede bacterial and leukocyte infiltration of the tissue. Some of these responses have been reported previously, such as the upregulation of vascular adhesion molecules and glycocalyx components, whereas other were completely unexpected, such as the compartmentalization of the acute-phase reaction and the massive shedding of vasculature-associated IgGs. Future work should explore how these early vascular events are coordinated, for example, by focusing on transcriptional and/or posttranslational mechanisms of proteome regulation.

There is no single sepsis model that is optimal for all research purposes. The use of animal models in research has been the subject of debate due to their obvious limitations in fully recapitulating the complex clinical picture of human sepsis. However, it is also undeniable that most of our knowledge of the molecular basis of sepsis has been derived from studies mainly using the LPS model (endotoxin driven sepsis), the CLP model (polymicrobial sepsis through cecal ligation and puncture), and models of monomicrobial sepsis using peritoneal or intravenous inoculation, like in this study. Monomicrobial models of sepsis are advantageous particularly for assessing the progression of experimental sepsis due to their robustness and reproducibility, making it easy to define a starting point for the time course and a reproducible amount of bacterial inoculate injected into the animals. This idea is in line with the focus of our study to establish molecular and temporal relationships between vascular dysfunction and organ dysfunction. Although the injection of a large bolus of bacteria to induce sepsis in mice might differ in many aspects from the relatively slower development of human sepsis, vascular dysfunction and glycocalyx remodeling in the mouse have many similarities to what occurs in humans (27).

Finally, the accumulating data suggest that sepsis entails a series of heterogeneous responses to systemic infection, with distinct molecular trajectories and disparate clinical outcomes. The integration of these data across compartments through COR-SPOTS has allowed us to identify markers that are informative of processes happening in other tissues or compartments in S. aureus sepsis. However, this strategy can be exploited in numerous cases. For example, identification of vascular markers leaking out to the plasma could potentially be measured using more sensitive and targeted approaches directly on clinical material. Additionally, construction of concise molecular readouts of the host response during systemic inflammation can be used to compare the impact of individual pathogens and/or virulence factors to the molecular heterogeneity of sepsis.

## MATERIALS AND METHODS

### Bacterial preparation and infection.

S. aureus strain USA300/TCH1516 was grown at 37°C in liquid cultures of Todd-Hewitt broth (THB; Difco) with agitation (200 rpm), and later incubated in 5 mL of fresh THB overnight. Roughly, 400 μL of the overnight culture was inoculated into 6 mL of fresh THB and incubated until an optical density of 600 nm (OD_600_) of 0.4 was reached. Bacteria were centrifuged, washed twice, and resuspended in phosphate-buffered saline (PBS). Next, 8- to 10-week-old C57BL/6 mice were infected intravenously through the retroorbital sinus with 5 × 10^7^ CFU of the bacterial culture in 100 μL PBS or just with 100 μL PBS in the control group. Animals were euthanized at 6 h, 12 h, or 24 h using isoflurane. One group of animals was immediately subjected to chemical biotinylation perfusions as described below, and another group was subjected to cardiac puncture to collect blood samples. After these procedures, multiple organs were collected from both groups (liver, kidney, heart, and spleen). All animals were housed in individual ventilated cages in vivaria approved by the Association for Assessment and Accreditation of Laboratory Animal Care at the School of Medicine, University of California (UC) San Diego. All experiments followed relevant guidelines and regulations consistent with standards and procedures approved by the UC San Diego Institutional Animal Care and Use Committee (protocol S99127 and S00227M).

### Systemic chemical perfusions.

Chemical perfusions were performed as reported previously (17). Briefly, mice were subjected to a median sternotomy, and the left ventricle of the heart was punctured with a 25-gauge butterfly needle (BD Vacutainer). A small cut was made in the right atrium to allow draining of perfusion solutions. All ice-cold perfusion reagents were infused using a perfusion pump (Fischer Scientific). Blood was washed out with PBS for 5 min at a rate of 5 mL/min. A solution containing 100 mM EZ-link sulfo-NHS-biotin (Thermo Fischer) in PBS (pH 7.4) was perfused at a rate of 3 mL/min for 10 min. Finally, a quenching solution (50 mM Tris-HCl [pH 7.4]) was perfused at 3 mL/min for 5 min. Control animals were perfused with PBS only.

### Organ preparations.

Collected organs were homogenized in a buffer containing 5 M urea, 0.25 M NaCl, and 0.1% SDS. Samples were briefly centrifuged for 5 min, and the supernatant was transferred to new tubes. Protein was quantified by a standard bicinchoninic acid (BCA) assay (Thermo Scientific) as per the manufacturer’s instructions. Samples were stored at −80°C until further analysis. In parallel, a piece of each organ was homogenized in 1 mL ice-cold PBS, and the samples were plated on Todd-Hewitt agar for CFU analysis.

### Blood chemistry and coagulation assays.

Whole blood was collected via cardiac puncture and placed in a procoagulant serum tube (BD Microtainer; no. 365967) for 4 h at room temperature. Serum was isolated by spinning the tubes at 2,000 × *g* and collecting the supernatant. All samples were frozen and thawed once before analysis. Blood chemistry parameters were measured on a Cobas 8000 automated chemistry analyzer (Roche) with a general coefficient of variance of <5%. All samples were frozen and thawed no more than two times before analysis. Blood coagulation factor assays were performed as described previously (28).

### Bacterial CFU counts.

Organs of interest were harvested at the indicated time points and placed in a 2-mL tube (Sarstedt; no. 72.693.005) containing 1 mL ice-cold PBS and 1.0-mm-diameter zirconia/silica beads (Biospec Products; no. 11079110z). Samples were homogenized using a MagNA Lyzer instrument (Roche) for 2 min at 6,000 rpm. Whole blood was collected via cardiac puncture and placed in an EDTA tube (BD Microtainer; no. 365974). An aliquot of each organ or blood sample was serially diluted in PBS and plated on Todd-Hewitt agar to enumerate CFU.

### Histological analysis.

Tissues were harvested at the indicated time points and fixed in 10% buffered formalin (Fischer Chemical) for 24 h, followed by submersion in 70% ethanol for at least 24 h. The samples were paraffin embedded, sectioned (3 μm), and stained with hematoxylin-eosin.

### Purification of biotinylated proteins.

Biotinylated proteins were isolated from the organ homogenates using a Bravo AssayMap platform and AssayMap streptavidin cartridges (Agilent). The cartridges were equilibrated with ammonium bicarbonate (50 mM; pH 8), and biotinylated samples were loaded. Nonbiotinylated proteins were removed by extensive wash with 8 M urea in 50 mM ammonium bicarbonate buffer (pH 8). Cartridges were further washed with rapid digestion buffer (Promega; rapid digestion buffer kit), and bound proteins were subjected to on-column digestion using a mass spectrometry-grade trypsin/Lys-C rapid digestion enzyme (Promega, Madison, WI) at 70°C for 2 h. Released peptides were desalted using AssayMap C_18_ cartridges (Agilent). Samples were stored at −20°C prior to DIA-SWATH-MS analysis.

### DIA-SWATH-MS.

DIA-SWATH-MS analysis was performed on a Q Exactive HF-X mass spectrometer (Thermo Fisher Scientific) coupled to an EASY-nLC 1200 ultra-high-performance liquid chromatography system (Thermo Fisher Scientific). Peptides were trapped on a precolumn (PepMap100 C_18_, 3 μm; 75 μm by 2 cm; Thermo Fisher Scientific) and separated on an EASY-Spray column (ES803; column temperature 45°C; Thermo Fisher Scientific). Equilibrations of columns and sample loading were performed as per the manufacturer’s guidelines. Solvent A (0.1% formic acid), and solvent B (0.1% formic acid and 80% acetonitrile) were used to run a linear gradient from 5% to 38% over 120 min at a flow rate of 350 nL/min. A DIA method was implemented using a schedule of 44 variable acquisition windows as reported previously (19). The mass range for MS1 was 350 to 1,650 *m/z* with a resolution of 120,000 and a resolution of 30,000 for MS2 with a stepped normalized collisional energy (NCE) of 25.5%, 27%, and 30%.

### DIA-SWATH-MS data analysis.

An *in silico* spectral library was generated for the reference Mus musculus proteome (EMBL-EBI RELEASE 2020_04, 22,295 entries) using deep neural networks as implemented in DIA-NN (v1.7.10) (20). For search space reduction, a list of previous MS-detectible mouse peptides were compiled from the Peptide Atlas Project (29) and from two published large-scale DIA proteomics studies (11, 30). The compiled library with a final number of 667,455 precursors was used for DIA data extraction with a protein *q* value of 0.01 and retention time (RT) profiling enabled. Data were normalized using the cyclic loess normalization method in the NormalyzerDE tool.

### Statistical analysis.

All statistical methods were implemented using Python 3.6.10. Proteomics results from the DIA-SWATH-MS analysis were filtered using a one-way analysis of variance (ANOVA) followed by a Benjamini-Hochberg procedure to control for a false discovery rate (FDR) of <0.10. Statistically significant identifications were further subjected to principal-component analysis (PCA). Proteins were given a standardized score using a Z-score normalization. Proteomics results were analyzed separately using Welch’s *t* test to generate volcano plots and heatmaps.

### Functional enrichment analysis.

A functional enrichment analysis of differentially abundant proteins was performed through Database for Annotation, Visualization, and Integrated Discovery (DAVID). DAVID was run using default settings with the threshold count of ≥2 and enrichment threshold (EASE) of ≤0.1 (31). Visualization of enriched Genome Ontology (GO) terms was produced using code adapted for treemap visualizations from the Web tool Revigo (32).

### Nonnegative matrix factorization.

Plasma DIA proteomics was analyzed using nonnegative matrix factorization (NMF). Following the pseudocode of Lee and Seung’s multiplicative update rule, the distance between the data set matrix (D) and the dot product of hypothetical partitions (W and H) was minimized using a coordinate descent algorithm (21). The two resulting matrices were constrained during the optimization to allow for better interpretability. First, the predictions in the matrix H must fall within the range 0.0 to the maximum observed log_2_(relative abundance). Second, the distributions in the W matrix must fall in the range 0.0 to 1.0 and must sum across signatures to 1.0. In order to determine the number of predicted proteome signatures, k, for each value k, 200 random initializations were made. The average Pearson correlation between the ground truth hierarchy learned from D and the weights hierarchy learned from W and average distance scores (‖D−WH‖F) are plotted for visual inspections at each k. Cluster number k was finally selected using the elbow selection method as well as a meaningful investigation of correlation and proteome signature content.

### Clustering.

**(i) Abundance clustering.** Significant proteins were grouped based on their relative abundances using k-means clustering. K selection was done through the visualization of distances to cluster centers across k values up to 20 and selection based on the elbow method (33).

**(ii) Functional Clustering.** Protein identifications were parsed through the medium confidence (Protein-protein associations (PPA), >0.4) STRING-DB to build preliminary networks based on physical or functional associations. These networks were subjected to the Louvain method for community detection, a modularity-based clustering algorithm. The Louvain clustering was implemented using the Python packages NetworkX and python-Louvain with default settings (34). To circumvent the heuristic property of the Louvain algorithm, ~1,000 parallel random starts were performed initially. Using the combined results from these 1,000 iterations, a matrix of cluster clarity was built to store the average number of times two proteins appear in the same community, divided by the number of iterations. Using this community clarity matrix, a k-means clustering was finally performed with k being the average number of communities found across the 1,000 runs.

**(iii) Cluster annotations.** The Jaccard similarity index was also used to determine the overlap of proteins contained between two clusters by quantifying the intersection over the union. Members of a ranked list of functional associations for each cluster were defined by using a hypergeometric test (SciPy v1.4.1). Using the Tau-b statistic from the Kendall rank correlation test (SciPy v1.4.1), functional terms between two clusters were compared by ranked enriched terms across GO terms, Reactome, and KEGG pathways.

### Higher-order molecular networking.

All significant protein identifications from all data sets (i.e., different time points, organs, and compartments) were parsed through the functional clustering pipeline described in the previous section. Each of these higher-order community nodes contained a variable number of proteins (ranging from just a few to hundreds of proteins), operating within similar biological processes. Finally, we computed pairwise Spearman correlations over all combinations of detected community nodes, which resulted in the detection of significant correlations that were used to define linking edges between the nodes. The Benjamini-Hochberg procedure was used to control for a false discovery rate (FDR) of <0.10. The multiedge was then constructed using three pieces of relational information. Contained in the multiedge are the scores for the proportion of significant pairwise Spearman correlations, the Jaccard similarity index, and the Tau-b statistics defined previously. Visualization and analysis of the network layers were conducted through Cytoscape (35).

### Data availability.

We provide the raw data on MassIVE, a community resource developed by the NIH-funded Center for Computational Mass Spectrometry online at https://massive.ucsd.edu/ProteoSAFe/dataset.jsp?task=e0e5dd67e3af4cdd8f51f06439c2ac29. We provide the code library in Python described in this work through Github online at https://github.com/LewisLabUCSD/CORSPOTS. We provide jupyter notebooks in Python used to generate our figures and analysis.
