# Supplementary figures and images for: Vascular Proteome Responses Precede Organ Dysfunction in a Murine Model of Staphylococcus aureus Bacteremia
Source: mSystems. 2022 Aug 1;7(4):e00395-22. doi: 10.1128/msystems.00395-22 (PMC9426442; doi:10.1128/msystems.00395-22)

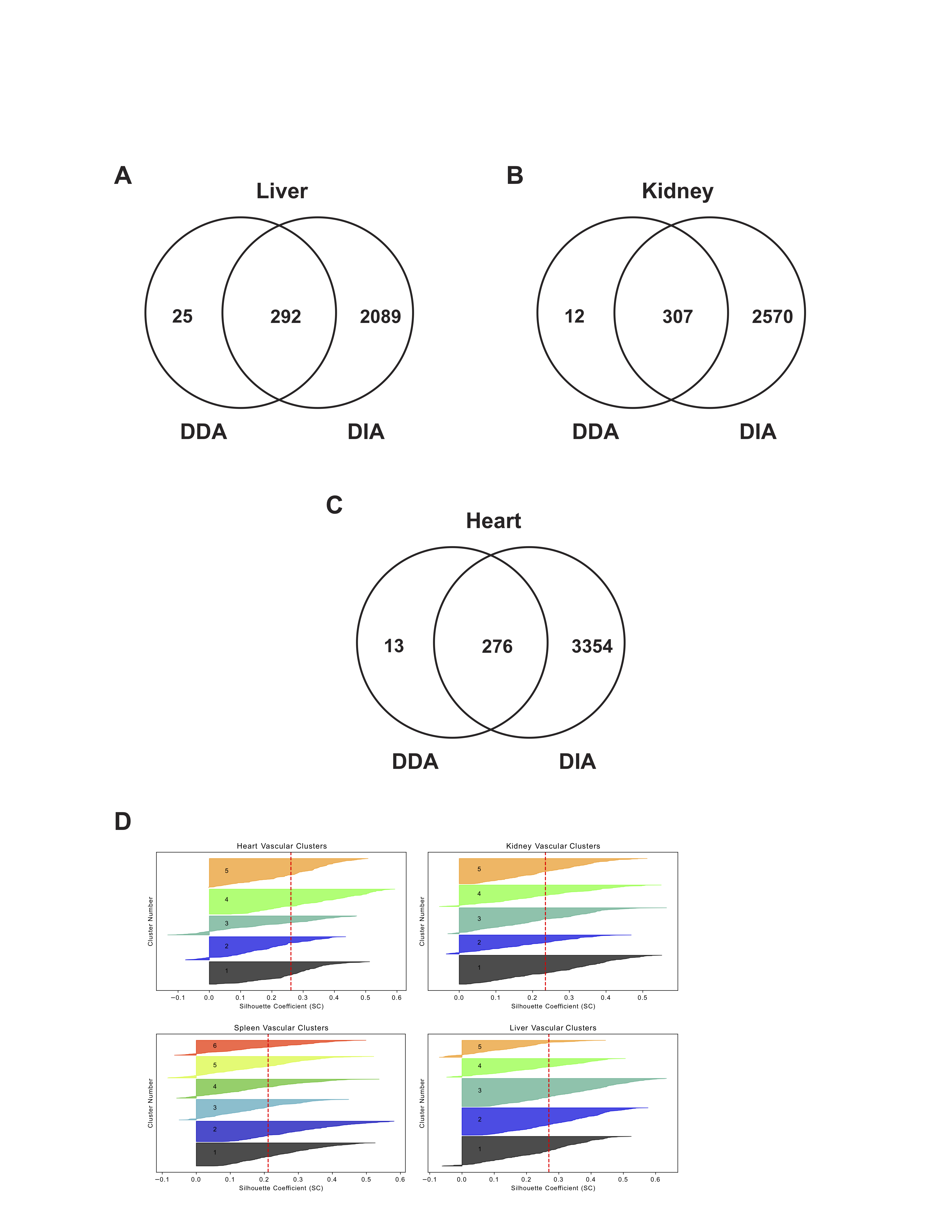

Supplement: FIG S1 [file msystems.00395-22-s0001.tif]

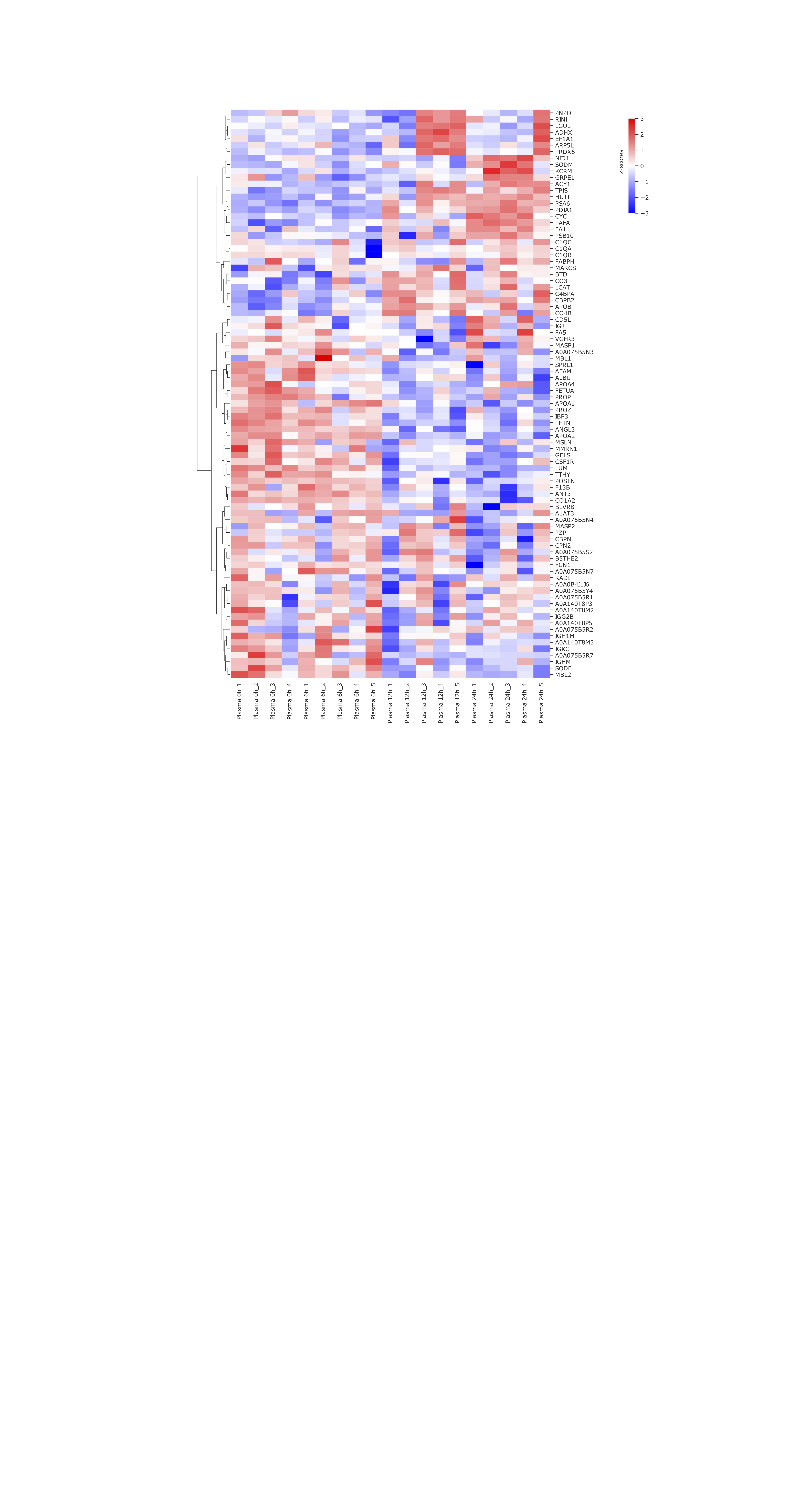

Supplement: FIG S2 [file msystems.00395-22-s0002.tif]

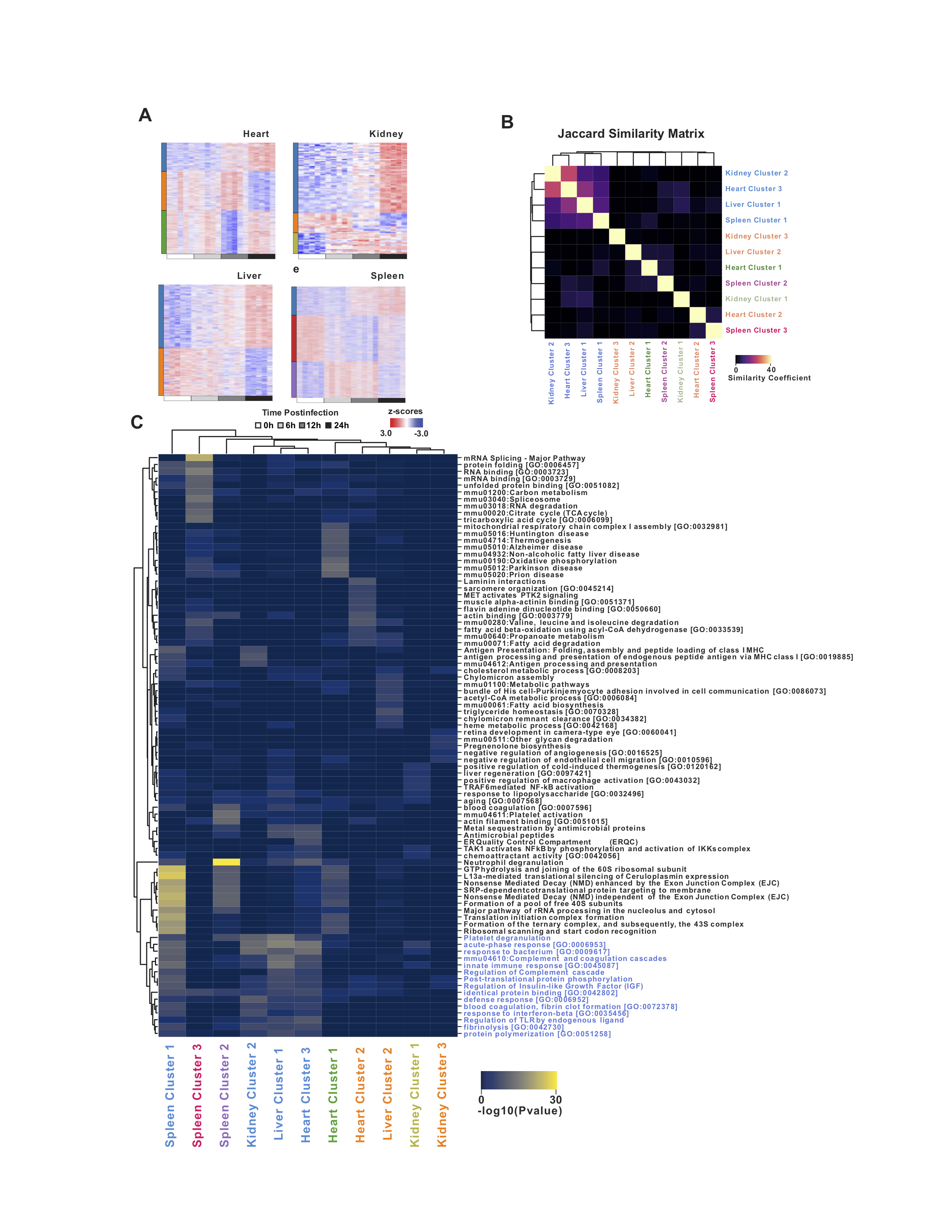

Supplement: FIG S3 [file msystems.00395-22-s0003.tif]
